# A Novel Index in the Prediction of Pneumonia Following Acute Ischemic Stroke

**DOI:** 10.3390/ijerph192215306

**Published:** 2022-11-19

**Authors:** Aleksandra Szylińska, Marta Bott-Olejnik, Paweł Wańkowicz, Dariusz Karoń, Iwona Rotter, Katarzyna Kotfis

**Affiliations:** 1 Department of Medical Rehabilitation and Clinical Physiotherapy, Pomeranian Medical University, 71-204 Szczecin, Poland; 2 Department of Neurology, Regional Specialist Hospital in Gryfice, 72-300 Gryfice, Poland; 3 Department of Anesthesiology and Intensive Therapy, Regional Specialist Hospital in Gryfice, 72-300 Gryfice, Poland; 4 Department of Anesthesiology, Intensive Therapy and Acute Intoxications, Pomeranian Medical University, 71-204 Szczecin, Poland

**Keywords:** acute ischemic stroke, pneumonia, COPD

## Abstract

Background: The aim of our study was to search for predictive factors and to develop a model (index) for the risk of pneumonia following acute ischemic stroke. Material and methods: This study is an analysis of prospectively collected data from the neurology department of a district general hospital in Poland, comprising 1001 patients suffering from an acute ischemic stroke. Based on the medical data, the formula for the prediction of pneumonia was calculated. Results: Multivariate assessment for pneumonia occurrence was performed using the new PNEUMOINDEX. The study showed a significant increase in pneumonia risk with an increasing PNEUMOINDEX (OR non-adjusted = 2.738, *p* < 0.001). After accounting for age and comorbidities as confounders, the effect of the Index on pneumonia changed marginally (OR = 2.636, *p* < 0.001). Conclusions: This study presents factors that show a significant association with the occurrence of pneumonia in patients with acute ischemic stroke. The calculated PNEUMOINDEX consists of data obtained at admission, namely NYHA III and IV heart failure, COPD, generalized atherosclerosis, NIHHS score on admission, and CRP/Hgb ratio, and shows high prediction accuracy in predicting hospital-acquired pneumonia in ischemic stroke patients.

## 1. Introduction

Hospital-acquired pneumonia is one of many serious complications that can occur following a stroke. Pneumonia considerably increases the risk of death (mainly in patients with acute ischemic stroke) and deteriorates patient outcomes, reducing the chances of a full recovery and frequently leading to disability [1,2]. While the incidence of pneumonia in stroke patients can range from 8% to 30% [3,4,5,6,7], it may reach even 44% in patients with chronic obstructive pulmonary disease (COPD) [7]. Therefore, it is important to provide an early prediction of the risk of hospital-acquired pneumonia in stroke patients.

Several risk factors have already been identified, such as age, male gender, comorbidities, and the severity of the stroke [8,9]. The occurrence of the stroke itself is associated with many well-studied risk factors, such as age, diabetes, smoking, dyslipidemia, and hypertension [10,11]. Recent studies suggest that anemia is also associated with high in-hospital mortality in stroke patients [12,13]. A study conducted in a hospital emergency department suggests a strong negative association between CRP and Hgb levels, increasing the incidence of hospitalization of the patients [14,15]. Therefore, in our study, we also decided to analyze the C-reactive protein-to-hemoglobin ratio.

Early recognition of patients with a high risk of developing pneumonia is essential, not only in the critical care unit, but also in every hospital ward admitting high-risk patients. Indeed, intensivists have created risk prediction models designed to predict pneumonia in stroke patients, demonstrating variable, but reasonable, degrees of sensitivity and specificity, including the A2DS2, ISAN, and PANTHERIS scores [16,17,18,19].

Investigating the relationships between medical history, symptoms on admission, and blood analyses is an important ongoing element throughout the treatment process [20]. Studies show that blood laboratory analyses influence 70% of medical decisions [21].

Our study aimed to search for predictive factors and to develop a model (index) for the risk of pneumonia following acute ischemic stroke.

## 2. Material and Methods

### 2.1. Study Population

This study is an analysis of prospectively collected data from the neurology department of a district general hospital in Poland between June, 2015, and March, 2018, comprising 1022 patients suffering from an acute ischemic stroke with symptom onset within 48 to 72 h. Exclusion criteria included incomplete laboratory results, no data regarding follow-up, and coexisting hematological disorders. Twenty-one subjects were excluded from the analysis; 1001 patients were further analyzed. The subjects were divided into two groups 774 patients without pneumonia and 227 patients with pneumonia.

### 2.2. Data Collection

From their medical records, information was collected regarding demographics (age, gender, BMI, smoking) and comorbidities (arterial hypertension, ischemic heart diseases, myocardial infarction, NYHA type III and IV, TIA, ischemic stroke, history of hemorrhagic stroke, acute chronic renal failure on admission, chronic dialysis, impaired insulin tolerance, diabetes, gout, extracardiac arteriopathy, COPD, atrial fibrillation, and carotid stenosis). Routine neurological examinations at admission and at discharge provided information regarding stroke severity, according to the National Institutes of Health Stroke Scale (NIHSS), and disability, according to the modified Rankin Scale. In addition, data regarding the results of routine laboratory tests (blood count, serum creatinine, C-reactive protein, troponin T, cholesterol, triglyceride, and liver function tests) were collected from their medical records.

Outcome data were obtained from medical records and by telephone at 30 and 90 days after the patient was discharged from the ward. Patient death was recorded as in-hospital mortality up to day 7. Information on subsequent death at the 1-year follow-up was obtained by telephone contact, and the mortality rates at 30 and 90 days and 1 year after stroke were calculated.

### 2.3. CRP and Hemoglobin

In our research, we decided to analyze the C-reactive protein-to-hemoglobin ratio [14]. From the medical records, we obtained information about the concentration of CRP and Hb in the blood on the day of admission of a patient with acute ischemic stroke. CRP/Hb was calculated based on the formula:

CRP/Hgb = C-reactive Protein [mg/L]:Hemoglobin [g/dL].(1)

### 2.4. Diagnosis of Pneumonia

The definition of pneumonia was based on the guidelines of the American Thoracic Society, where it is described as the presence of typical changes in chest radiography with at least two of the following: fever, leukocytosis (white blood cell WBC > 12 G/L) or leukopenia (WBC < 4 G/L), or expectoration of pus sputum [22].

### 2.5. Ethical Statement

Prospective data collection was performed, according to the guidelines of the Declaration of Helsinki. We obtained the approval of the Bioethical Committee of the Pomeranian Medical University in Szczecin, Poland, decision no. KBE-0012/84/03/19. The requirement to obtain informed consent was waived, as the study was performed as a routine clinical process of the diagnosis and treatment performed in every patient admitted to the hospital. Patient confidentiality was ensured with the use of anonymized data.

### 2.6. Statistical Analysis

Statistical analysis was performed using specialized software (StatSoft, Inc., Tulsa, OK, USA). Descriptive statistics, based on means, medians, standard deviations, counts, and percentages, were used to show the characteristics of the group. Continuous data was compared using Mann–Whitney U tests, and qualitative data using Chi-square or Chi-square with Yates’s correction tests. Regression analysis was performed to look for risk factors affecting the incidence of pneumonia. The formula for an optimal prediction of pneumonia was calculated and presented as PNEUMOINDEX. A receiver operating characteristic (ROC) analysis was performed to determine the diagnostic value of the PNEUMOINDEX and its components for predicting pneumonia. Multivariate logistic regression analysis, adjusted for age and comorbidities for the incidence of pneumonia, was performed. Differences were regarded as statistically significant at *p* < 0.05.

## 3. Results

Table 1 presents the baseline data collected on the day of hospital admission. In the group of stroke patients diagnosed with pneumonia, greater age (*p* < 0.001), higher RANKIN (*p* < 0.001) and higher NIHHS (*p* < 0.001) scores were observed. The patients with pneumonia on admission were more likely to have ischemic heart diseases (*p* < 0.001), myocardial infarction (*p* = 0.033), NYHA III, and IV heart failure (*p* < 0.001), history of ischemic stroke (*p* = 0.001), chronic renal failure (*p* < 0.001), impaired insulin tolerance (*p* = 0.031), diabetes on insulin (*p* = 0.002), extracardiac arteriopathy (*p* < 0.001), and atrial fibrillation (*p* < 0.001), COPD (*p* < 0.001).

Analysis of laboratory data performed on the day of admission showed higher levels of glycemia (*p* = 0.004), leukocytes (*p* < 0.001), neutrophils (*p* < 0.001), creatinine (*p* < 0.001), CRP (*p* < 0.001), and troponin T (*p* < 0.001) in the patients with pneumonia. and lower levels of lymphocytes (*p* < 0.001), hemoglobin (*p* < 0.001), cholesterol (*p* < 0.001), and triglyceride (*p* < 0.001). These results are presented in Table 2.

Table 3 presents the outcome data for the admitted ischemic stroke patients. Those with pneumonia during their hospitalization had a significantly higher risk of mortality at 7 days (*p* < 0.001), 30 days (*p* < 0.001), 90 days (*p* < 0.001), and 1 year (*p* < 0.001).

A univariate logistic regression analysis was performed for pneumonia, as shown in Table 4. In stroke patients, the analysis showed an association between multiple factors at admission and pneumonia during the hospital stay (Table 4). This prompted the researchers to perform further analyses.

In the search for factors determining the occurrence of pneumonia during the hospital stay of ischemic stroke patients, multiple multivariate regression models were performed, clustering all statistically significant parameters from the univariate analysis. These analyses in the study group resulted in a model with factors that had the highest influence on the occurrence of pneumonia. The model is presented in Table 5. The analysis showed an association between pneumonia and heart failure on NYHA III and IV scale (OR = 4.56, *p* < 0.001), COPD (OR = 2.66, *p* < 0.001), extracardiac arteriopathy (OR = 3.06, *p* < 0.001), NIHHS scale at discharge (OR = 1.15, *p* < 0.001), and CRP/HGB ratio (OR = 1.17, *p* < 0.001).

Based on the obtained factors in Table 5, with the highest association with pneumonia, a predictive index for pneumonia was created. The PNEUMOINDEX is predictive of the occurrence of pneumonia during the hospital stay, and was calculated using the formula:

PNEUMOINDEX = (1.516 × NYHA III & IV) + (0.978 × COPD) + (1.117 × extracardiac arteriopathy) + (0.141 × NIHHS on the day of admission) + (0.153 × CRP/HGB)(2)

**Figure 1:**
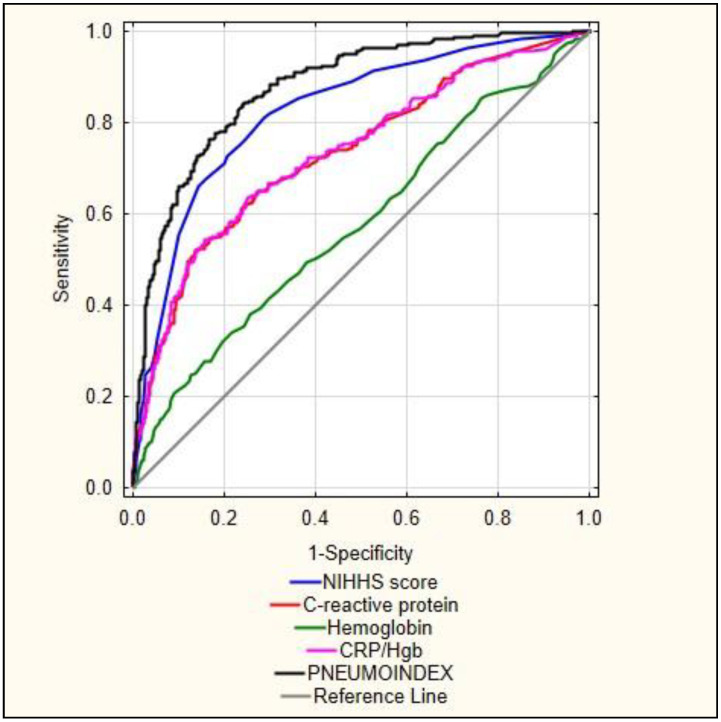
ROC analysis curves for pneumonia. Legend: NIHSS—National Institutes of Health Stroke Scale, CRP/HGB—C-reactive protein-to-hemoglobin ratio.

ROC curve analysis for the quantitative data: NIHHS score on the day of admission, CRP and Hb values every day, CRP/Hb and calculated PNEUMOINDEX value is presented in Figure 1. Table 6 presents the results of the ROC curve. This analysis showed a very high area under the curve for PNEUMOINDEX (AUC = 0.876, *p* < 0.001). PNEUMOINDEX obtained a significantly higher predictive value than each parameter separately.

Multivariate assessment for pneumonia occurrence was performed using the newly calculated PNEUMOINDEX. The study showed a significant increase in pneumonia risk with an increasing PNEUMOINDEX (OR non-adjusted = 2.738, *p* < 0.001). After accounting for age and comorbidities as confounders, the effect of the Index on pneumonia changed marginally (OR = 2.636, *p* < 0.001) Table 7.

## 4. Discussion

In current clinical practice, assessing the risk of pneumonia in patients with acute ischemic stroke remains a major challenge. Therefore, in this study we attempted to create a simple universal index that would be helpful for clinicians to quickly detect/identify patients with acute ischemic stroke having the highest risk of pneumonia.

Indeed, our study reports a relatively high risk of pneumonia in stroke patients. The research indicated that the incidence of pneumonia in stroke patients ranged from 7.1% to over 30%. Our research showed that over 20% of patients developed pneumonia, which fell within the ranges reported by other authors. A study by Finlayson et al., reported that 587 out of 8251 (7.1%) patients developed stroke-associated pneumonia [6]. In a more recent study by Liu et al., the authors were able to propose a care bundle that included interventions aimed at reducing the incidence of pneumonia after stroke, such as the utilization of the following tools: for SAP risk screening, dysphagia screening, and adequate rehabilitation; for feeding modification, oral care, airway management, and position management. They also proposed utilizing the nursing techniques of traditional Chinese medicine. The authors reported an initial incidence of 37.2% in the pre-implementation period and 14% in the post-implementation group (*p* = 0.025) [23].

Our study showed that an index meeting the above assumptions could be provided by the pneumonia index we developed. This index included the following variables: NYHA III and IV heart failure, COPD, generalized atherosclerosis, NIHHS scale, and CRP/hemoglobin ratio. Patients with CHF (chronic heart failure) are thought to have twice the risk of pneumonia compared with age- and sex-matched subjects in the general population, while survival from pneumonia is lower in patients with coexisting CHF than in those without CHF. Conversely, pneumonia increases the risk of worsening CHF and is often considered a factor in cardiopulmonary decompensation leading to hospitalization or death. An analysis by Shen et al. revealed that 6.3% of patients participating in the PARADIGM-HF trial, and 10.6% of patients participating in the PARAGON-HF trial, developed pneumonia. In addition, the researchers also found that patients with heart failure with preserved ejection fraction (HFpEF) were at the highest risk of developing pneumonia [24,25,26].

COPD is one of the most common medical conditions, and is also a risk factor for developing pneumonia. Numerous clinical studies of pneumonia, that included outpatient, inpatient, and ICU cohorts, indicated that COPD is a frequently reported comorbid condition [27,28,29,30,31]. Patients with COPD had more severe pneumonia that led to unavoidable hospitalization and had a poorer prognosis [32,33,34] than those without. In the first year after COPD diagnosis, patients were 16 times more likely to develop pneumonia compared to patients without COPD. The incidence of out-of-hospital pneumonia was 22.4 events per 1000 person-years within 10 years of COPD diagnosis and was 50% higher in those classified as having severe COPD [35,36]. Hospitalized patients with COPD and coexisting pneumonia had significantly higher 30- and 90-day mortality rates than patients without COPD [33].

Increased disability, impaired consciousness, impaired awareness, or the presence of pseudobulbar syndrome generate higher NIHSS scores and are associated with a propensity for aspiration and hypostatic pneumonia. The results of other studies also support our observations that high NIHSS scale scores increase the risk of pneumonia in patients with acute ischemic stroke [37,38,39].

To the best of our knowledge, the relationship between serum CRP and Hb concentration has not been previously determined for stroke unit patients. The results of this study indicated that blood Hb and CRP concentrations were strongly associated with each other. The mean Hb concentration was significantly lower in patients with high CRP. In the study by Santos-Silva et al., a significant association between Hb and CRP was observed, especially in patients with respiratory diagnoses [15]. The CRP/Hb ratio included in the PNEUMOINDEX significantly increased the predictive value of this Index.

Comparing different models to predict pneumonia in a meta-analysis, Zhang et al. demonstrated that the AIS–SAP model showed the highest clinical value for assessing the prediction of pneumonia after AIS. The authors described that, for predicting in-hospital pneumonia after AIS, the area under the ROC curve for AIS–APS was 0.79, for A2DS2 score AUC was 0.74, and for the ISAN score AUC was 0.76 to [40]. In our study, the area under the PNEUMOINDEX curve was 0.87, making it an important, easily obtainable tool for the prediction of pneumonia after acute ischemic stroke that may be useful in everyday clinical practice.

## 5. Limitations

This study is not without limitations. It is a single-center observational series, so it would be useful to extend the research into a multi-center study. Further prospective studies with a larger sample size are needed.

## 6. Conclusions

This study presents factors that show a significant association with the occurrence of pneumonia in patients with acute ischemic stroke. The calculated PNEUMOINDEX consists of data obtained at admission, namely NYHA III & IV heart failure, COPD, generalized atherosclerosis, NIHHS score on admission, and CRP/Hgb ratio, and shows high accuracy in predicting hospital-acquired pneumonia in ischemic stroke patients.

## Figures and Tables

**Table 1:** Demographic data and comorbidities for the admitted ischemic stroke patients.

	No Pneumonia (n = 774)	Pneumonia (n = 227)	*p*
**Demographic data**			
Age [years], mean ± SD; Me	70.55 ± 12.29; 70.0	77.09 ± 10.35; 79.0	<0.001
Gender [male], n (%)	405 (52.33%)	118 (51.98%)	0.927
BMI [kg/m^2^], mean ± SD; Me	26.88 ± 4.74; 25.9	26.56 ± 4.58; 26.3	0.786
Smoking, n (%)	320 (41.34%)	97 (42.73%)	0.709
**Comorbidities**			
Arterial hypertension, n (%)	664 (85.79%)	205 (90.31%)	0.077
Ischemic heart diseases, n (%)	167 (21.58%)	91 (40.09%)	<0.001
Myocardial infarction, n (%)	74 (9.56%)	33 (14.54%)	0.033
NYHA 3 & 4, n (%)	16 (2.07%)	26 (11.45%)	<0.001
TIA, n (%)	146 (18.86%)	53 (23.35%)	0.136
History of ischemic stroke, n (%)	154 (19.90%)	68 (29.96%)	0.001
History of hemorrhagic stroke, n (%)	19 (2.45%)	5 (2.20%)	0.827
Acute renal failure on admission, n (%)	5 (0.65%)	4 (1.76%)	0.117
Chronic renal failure, n (%)	90 (11.63%)	53 (23.35%)	<0.001
Chronic dialysis, n (%)	1 (0.13%)	1 (0.44%)	0.354
Impaired insulin tolerance, n (%)	31 (4.01%)	17 (7.49%)	0.031
Diabetes on oral medications, n (%)	151 (19.51%)	54 (23.79%)	0.160
Diabetes on insulin, n (%)	86 (11.13%)	43 (18.94%)	0.002
Gout, n (%)	45 (5.81%)	20 (8.81%)	0.107
Extracardiac arteriopathy, n (%)	290 (37.47%)	164 (72.25%)	<0.001
COPD, n (%)	53 (6.85%)	42 (18.50%)	<0.001
Atrial fibrillation, n (%)	192 (24.84%)	101 (44.49%)	<0.001
Carotid artery stenosis, n (%)	61 (36.53%)	26 (52.00%)	0.051
**Scale at admission**			
Rankin score, mean ± SD; Me	9.20 ± 6.52; 7.0	18.74 ± 8.05; 20.0	<0.001
NIHSS, mean ± SD; Me	2.73 ± 1.52; 2.0	4.43 ± 1.08; 5.0	<0.001

Legend: n—number of patients, SD—standard deviation, Me-median, BMI—body mass index, NYHA—New York Heart Association, TIA—transient ischemic attack, COPD—chronic obstructive pulmonary disease, NIHSS—National Institutes of Health Stroke Scale.

**Table 2:** Laboratory data for the admitted ischemic stroke patients.

Variable	No Pneumonia (n = 774)			Pneumonia (n = 227)			*p*
	Mean	Me	±SD	Mean	Me	±SD	
Glycemia (mg/dl)	141.56	123.00	61.85	147.99	135.00	56.94	0.004
Leucocyte count (×10^9^/L)	9.45	8.89	3.37	11.02	9.71	5.38	<0.001
Neutrophil count (×10^9^/L)	6.61	5.82	4.35	8.26	7.18	4.93	<0.001
Lymphocyte count (×10^9^/L)	2.10	1.89	2.48	1.85	1.58	1.62	<0.001
Platelet count (×10^9^/L)	239.92	228.00	81.93	239.83	225.00	104.37	0.649
Hemoglobin	13.95	14.00	1.75	13.39	13.60	2.01	<0.001
Creatinine	1.04	0.91	0.60	1.20	1.01	0.71	<0.001
C-reactive protein	10.56	2.60	25.05	46.34	16.67	71.12	<0.001
Aspartate aminotransferase	24.26	20.00	14.42	36.96	21.00	108.93	0.701
Alanine aminotransferase	22.70	18.00	14.01	29.21	17.00	72.94	0.133
Cholesterol	199.48	195.00	53.48	171.02	167.00	49.79	<0.001
Triglycerides	146.26	122.00	91.21	121.54	105.00	63.92	<0.001
Troponin T	27.52	10.00	89.47	58.09	21.98	137.19	<0.001
CRP/Hgb	0.83	0.19	2.07	3.73	1.15	5.98	<0.001

Legend: n—number of patients, SD—standard deviation, Me—median, CRP/HGB—C-reactive protein-to-hemoglobin ratio.

**Table 3:** Outcome data for the admitted ischemic stroke patients.

Variables		No Pneumonia (n = 774)	Pneumonia (n = 227)				*p*
		mean ± SD; Me/n (%)	mean ± SD; Me/n (%)	OR	CI − 95%	CI + 95%	
Hospitalization time (days)		9.34 ± 4.05; 8.0	15.43 ± 11.56; 13.0	1.168	1.131	1.206	<0.001
NIHSS at discharge		7.21 ± 10.02; 3.0	23.61 ± 13.75; 20.0	1.096	1.082	1.111	<0.001
Rankin score at discharge		1.99 ± 2.02; 1.0	4.79 ± 1.39; 5.0	2.090	1.884	2.317	<0.001
Mortality until day 7		36 (4.65%)	32 (14.10%)	3.364	2.037	5.556	<0.001
Mortality until day 30		63 (8.14%)	104 (45.81%)	9.542	6.612	13.771	<0.001
Mortality until day 90		91 (11.76%)	151 (66.52%)	14.912	10.489	21.202	<0.001
Mortality until year 1		135 (17.44%)	169 (74.45%)	13.792	9.706	19.598	<0.001
Outcome	In-hospital death	38 (4.91%)	66 (29.07%)	7.940	5.144	12.255	<0.001
	Discharged home	510 (65.89%)	74 (32.60%)	0.250	0.183	0.343	<0.001
	Nursing home	44 (5.68%)	48 (21.15%)	4.449	2.864	6.912	<0.001
	Rehabilitation facility	172 (22.22%)	33 (14.54%)	0.595	0.397	0.894	0.012
	Another ward	10 (1.29%)	6 (2.64%)	2.074	0.746	5.770	0.162

Legend: n—number of patients, SD—standard deviation, Me—median, OR—odds ratio, CI—confidence interval, NIHSS—National Institutes of Health Stroke Scale.

**Table 4:** Regression analysis for pneumonia.

Variables	*p*	OR	CI − 95%	CI + 95%
**Demographic data**				
Age [years]	<0.001	1.051	1.036	1.066
Gender [male]	0.927	0.986	0.734	1.326
BMI [kg/m^2^]	0.367	0.985	0.954	1.018
Smoking	0.709	1.059	0.785	1.428
**Co-morbidities**				
Arterial hypertension	0.079	1.544	0.952	2.504
Ischemic heart diseases	<0.001	2.432	1.773	3.336
Myocardial infarction	0.034	1.609	1.036	2.498
Heart failure III and IV NYHA	<0.001	6.128	3.225	11.644
TIA	0.137	1.310	0.917	1.871
History of ischemic stroke	0.001	1.722	1.232	2.405
History of hemorrhagic stroke	0.827	0.895	0.330	2.424
Acute renal failure on admission	0.133	2.759	0.735	10.361
Chronic renal failure	<0.001	2.315	1.586	3.378
Chronic dialysis	0.384	3.436	0.214	55.147
Impaired insulin tolerance	0.033	1.940	1.053	3.575
Diabetes on oral medications	0.161	1.288	0.904	1.834
Diabetes on insulin	0.002	1.867	1.251	2.787
Gout	0.110	1.565	0.904	2.710
Extracardiac arteriopathy	<0.001	4.345	3.139	6.013
Atrial fibrillation	<0.001	2.426	1.782	3.302
COPD	<0.001	3.088	1.997	4.776
Carotid artery stenosis	0.052	1.883	0.995	3.563
**Scale at admission**				
Rankin score	<0.001	1.175	1.148	1.203
NIHSS	<0.001	2.436	2.112	2.809
**Laboratory data**				
Glycemia 0 (mg/dl)	0.164	1.002	0.999	1.004
Leucocyte count (×10^9^/L)	<0.001	1.094	1.055	1.135
Neutrophil count (×10^9^/L)	<0.001	1.088	1.047	1.131
Lymphocyte count (×10^9^/L)	0.064	0.854	0.724	1.009
Platelet count (×10^9^/L)	0.990	1.000	0.998	1.002
Hemoglobin	<0.001	0.848	0.783	0.919
Creatinine	0.003	1.444	1.134	1.838
C-reactive protein	<0.001	1.020	1.015	1.025
Aspartate aminotransferase	0.295	1.004	0.996	1.013
Alanine aminotransferase	0.281	1.004	0.997	1.011
Cholesterol	<0.001	0.989	0.985	0.992
Triglycerides	<0.001	0.996	0.993	0.998
Troponin T	0.002	1.002	1.001	1.004
CRP/HGB	<0.001	1.266	1.198	1.339

Legend: n—number of patients, SD standard deviation, Me—median, OR—odds ratio, CI—confidence interval, BMI—body mass index, NYHA—New York Heart Association, TIA—transient ischemic attack, COPD—chronic obstructive pulmonary disease, NIHSS—National Institutes of Health Stroke Scale, CRP/HGB—C-reactive protein-to-hemoglobin ratio.

**Table 5:** Regression model for patients with pneumonia.

	*p*	OR	CI − 95%	CI + 95%
NYHA III & IV	<0.001	4.555	1.979	10.487
COPD	<0.001	2.659	1.540	4.593
Extracardiac atherosclerosis	<0.001	3.055	2.060	4.530
NIHHS score (points)	<0.001	1.152	1.122	1.181
CRP/HGB	<0.001	1.166	1.101	1.234

Legend: OR—odds ratio, CI—confidence interval, NYHA—New York Heart Association, COPD—chronic obstructive pulmonary disease, NIHSS—National Institutes of Health Stroke Scale, CRP/HGB—C-reactive protein-to-hemoglobin ratio.

**Table 6:** Details of the receiver operating characteristics (ROC) analysis for pneumonia.

	AUC	AUC − 95%	AUC + 95%	*p*
PNEUMOINDEX	0.876	0.851	0.902	<0.001
NIHHS score	0.825	0.794	0.856	<0.001
CRP/Hgb	0.735	0.696	0.775	<0.001
C-reactive protein	0.734	0.695	0.773	<0.001
Haemoglobin	0.577	0.534	0.621	<0.001

Legend: AUC—Area under the ROC Curve, OR—odds ratio, CI-confidence interval, NIHSS—National Institutes of Health Stroke Scale, CRP/HGB—C-reactive protein-to-hemoglobin ratio.

**Table 7:** Multivariate logistic regression for pneumonia using PNUEMOINDEX.

	*p*	OR	CI − 95%	CI + 95%
PNEUMOINDEX	<0.001	2.738	2.381	3.149
PNEUMOINDEX *	<0.001	2.639	2.292	3.037
PNEUMOINDEX **	<0.001	2.636	2.281	3.047

Legend: OR—odds ratio, CI—confidence interval. Notes: * adjusted by age, ** adjusted by age and comorbidities not included in PNEUMOINDEX.

## Data Availability

Data sharing is not applicable.
